# Primary delayed gastric emptying after pylorus-resecting pancreatoduodenectomy: A matched-pair comparison of Roux-en-Y vs. Billroth-II reconstruction

**DOI:** 10.1016/j.sopen.2024.10.005

**Published:** 2024-10-31

**Authors:** Felix O. Hofmann, Victoria S. Engelstädter, Ughur Aghamaliyev, Mathilda M. Knoblauch, Elise Pretzsch, Maximilian Weniger, Jan G. D'Haese, Bernhard W. Renz, Jens Werner, Matthias Ilmer

**Affiliations:** a Department of General, Visceral and Transplantation Surgery, Ludwig-Maximilians-University Hospital Munich, Marchioninistrasse 15, 81377 Munich, Germany; b German Cancer Consortium (DKTK), Partner Site Munich, German Cancer Research Center (DKFZ), Heidelberg, Germany; c Department of General, Visceral and Vascular Surgery, Agatharied Hospital, Hausham, Germany

**Keywords:** Pancreatic ductal adenocarcinoma, Pylorus-resecting partial pancreatoduodenectomy, Delayed gastric emptying, Roux-en-Y reconstruction, Billroth-II reconstruction

## Abstract

**Background:**

After pylorus-resecting pancreatoduodenectomy (PrPD), delayed gastric emptying (DGE) might partially be attributed to biliary reflux. We investigated whether the incidence of primary DGE is reduced after Roux-en-Y instead of Billroth-II reconstruction.

**Methods:**

Patients undergoing PrPD from 2016 to 2019 at a high-volume center were identified. Excluding causes of secondary DGE, we matched patients with Roux-en-Y and Billroth-II reconstruction in a 1:2 ratio and compared primary DGE.

**Results:**

In 24 vs. 48 (Roux-en-Y vs. Billroth-II) patients, DGE (grade B/C) incidence (20.8 % vs. 18.8 %; *P* = 1.000), nasogastric tube requirement (median 2 vs. 2 days; *P* = 0.844) and time to solid food intake (7 vs. 7 days; *P* = 0.933) were comparable. Univariable logistic regression showed no association between DGE and Roux-en-Y reconstruction (OR 1.47; *P* = 0.524), in contrast to age (1.08; *P* = 0.030) and pancreatic biochemical leak (4.98; *P* = 0.007).

**Conclusions:**

Primary DGE did not differ between Roux-en-Y and Billroth-II reconstruction after PrPD. Instead, age and postoperative pancreatic biochemical leak were associated with higher DGE risk.

## Highlights

• Comparison of Roux-en-Y vs. Billroth-II reconstruction after PrPD
• Similar incidence of delayed gastric emptying (DGE), regardless of reconstruction
• Age and pancreatic biochemical leakage were associated with increased risk of DGE

## Introduction

Pancreatic ductal adenocarcinoma (PDAC) is projected to become the second leading cause of cancer-related death by 2030, driven by its rising incidence and persistently high mortality rates [1]. Resection remains the only curative option and should be attempted in combination with systemic treatment whenever feasible [2,3].

Despite significant reductions in mortality over the past decades, the morbidity following pancreatic surgery remains substantial. Delayed gastric emptying (DGE), as defined by the International Study Group of Pancreatic Surgery (ISGPS), involves the requirement of a nasogastric tube (NGT), intolerance of solid food, persistent vomiting, and the need of prokinetic medication [4]. DGE affects 20 to 80 % of patients [5,6], prolonging hospital stays, increasing costs, and diminishing quality of life [7,8].

In cases of pancreatic head tumors, both pylorus-resecting and pylorus-preserving partial pancreatoduodenectomy are established procedures [3]. Some studies advocate for preserving the pylorus as long as long-term effects of its resection remain unclear [9,10]. However, tumor infiltration can necessitate resection.

Theoretically, removing the pylorus, a potential mechanical obstacle, could lower DGE rates [11]. While some studies support this hypothesis [12,13], others do not [9,14], and a recent meta-analysis failed to provide conclusive evidence [15]. These inconsistent findings may be due to various confounders, such as technical differences (e.g., pylorus dilatation, reconstruction method), postoperative effects (e.g., vagotomy-induced atony, gastric dysrhythmia, cytokine dysfunction), heterogeneous study populations, or complications leading to secondary DGE [11,[16], [17], [18], [19]].

Biliary reflux is a potential confounder in patients undergoing pylorus-resecting partial pancreatoduodenectomy (PrPD) followed by (single-looped, omega-shaped) Billroth-II reconstruction. Reflux from the pancreaticobiliary jejunal loop into the stomach can cause DGE-like symptoms. We hypothesized that these symptoms could be reduced by Roux-en-Y reconstruction instead of Billroth-II reconstruction. This hypothesis is supported by findings from distal gastrectomy procedures, where nausea, vomiting and NGT requirements were reduced after Roux-en-Y instead of Billroth-II reconstruction [20].

This single-center, retrospective, matched-pair analysis compares the frequency and severity of primary DGE following PrPD with either Roux-en-Y or Billroth-II reconstruction.

## Methods

#### Study design and patients

This study is a retrospective, single-center analysis conducted at the Department of General, Visceral and Transplantation Surgery, Ludwig-Maximilians-University (LMU) Hospital, Munich, Germany, a high-volume tertiary referral center. We screened all patients who underwent pancreatic surgery between January 1, 2016, and December 31, 2019, for eligibility. This period was chosen to ensure highly standardized surgical and perioperative care and to avoid overlap with the prospective registry trial *PyloResPres*, which began recruitment at the end of 2019 [21].

Patients who underwent PrPD were identified. Records with missing values were excluded. We also excluded patients who underwent extended surgery (e.g., resection and reconstruction of the hepatic artery, hemicolectomy, total pancreatectomy, total or subtotal gastrectomy, or other multivisceral resections). Patients with non-surgical complications requiring prolonged intensive care unit (ICU) treatment associated with extended parenteral or enteral feeding were also excluded. Given that intraabdominal complications can cause secondary DGE and could obscure the relationship between reconstruction method and DGE due to potential biliary reflux [16,17,19,22], patients with intraabdominal complications graded Clavien-Dindo IIIa or higher were excluded [23]. Patients with postoperative pancreatic biochemical leakage, as defined by the ISGPS, were included [24].

This study was approved by the institutional review board of the medical faculty of the LMU University (22-0951) and registered at the Clinical Study Center of the LMU University Hospital (100230). Data screening was performed by one of the authors (FOH) under confidentiality obligations. Prespecified patient parameters were irreversibly anonymized before conducting further analyses.

### Data analysis

Patients who underwent PrPD followed by Roux-en-Y reconstruction were matched in a 1:2-ratio with those who underwent PrPD followed by Billroth-II reconstruction. Matching was performed using a propensity score that considered sex, age, ASA category, BMI, histopathology (malignant or benign) and postoperative pancreatic fistula (POPF) as defined by the ISGPS [18,24].

Baseline parameters and outcomes of patients with Roux-en-Y and Billroth-II reconstruction were compared. The primary outcome was DGE as defined by the ISGPS [4]. For further analysis, DGE was classified as either clinically non-relevant (no DGE or grade A) or relevant (grade B or C). Secondary outcomes included the duration of NGT use, time to tolerance of solid food, length of hospital stay, and 30-day readmission rate.

### Surgical procedures and perioperative care

Open partial pancreatoduodenectomy and perioperative care were highly standardized. The decision to resect the pylorus was made by the operating surgeon based on the suspicion of pyloric infiltration, pancreatic serosal invasion, or enlarged perigastric lymph nodes. The stomach was then divided approximately 2 cm proximal to the pyloric ring, while preserving the gastric vessels along both curvatures. The pancreas was transected at the plane of the superior mesenteric vein, and frozen section analysis performed routinely. Portal vein resection and reconstruction was conducted when necessary, and standard lymphadenectomy was performed in cases of suspected or known malignancy. After resection, the choice of reconstruction method – either Roux-en-Y or Billroth-II – was at the surgeon's discretion.

For the single-looped, omega-shaped Billroth-II reconstruction, the proximal jejunal stump was transposed through the mesocolon. An end-to-side, double-layer, duct-to-mucosa, dunking pancreaticojejunostomy and an end-to-side, single-layer hepaticojejunostomy were performed. Following antecolic transposition, an end-to-side, double-layer gastrojejunostomy was constructed approximately 50 cm distal to the hepaticojejunostomy. Depending on the surgeon's preference, a side-to-side, double-layer Braun enteroenterostomy was added. All anastomoses were hand-sewn using synthetic absorbable monofilament atraumatic sutures (PDS 5-0 or 4-0).

For the Roux-en-Y reconstruction, the jejunum was separated between the second and third loop. The proximal end of the second jejunal loop was transposed through the mesocolon, and the pancreaticojejunostomy and hepaticojejunostomy were performed as previously described. After antecolic transposition of the proximal end of the third jejunal loop, an end-to-side, double-layer gastrojejunostomy was constructed. Approximately 50 cm distal to the gastric anastomosis, an end-to-side, double-layer Roux-en-Y Braun jejunojejunostomy was performed.

The standard protocol included placing two closed-system drainages near the pancreaticojejunostomy and hepaticojejunostomy. In patients with soft pancreatic tissue or a small pancreatic duct, octreotide was applied subcutaneously three times per day for one week. All patients received daily standard doses of proton-pump inhibitors for three months postoperatively. Pancreatic enzyme levels in the intraabdominal drainages were measured routinely, and the drainages were removed when no enzymatic activity was detected or when fluid production ceased. If clinical or laboratory signs of infection appeared, computed tomography was performed, and relevant intraabdominal collections were drained by interventional radiology. Patient-controlled epidural analgesia was supplemented with intravenous medication when necessary.

NGT were removed either at the end of the operation, or postoperatively when reflux was low and there was no nausea or vomiting. If vomiting occurred without a NGT in place, replacement was considered on a case-by-case basis: For alert and attentive patients with moderate nausea, antiemetic and prokinetic medications were administered. Elderly patients, those who were unalert or inattentive, or those with severe nausea or recurrent vomiting had a new NGT inserted.

If significant reflux via the NGT persisted for >48 h postoperatively, laxatives were administered. In patients where reflux continued despite established gastrointestinal passage, intravenous metoclopramide and erythromycin were initiated. Persistent reflux prompted the consideration of computed tomography to exclude intraabdominal fluid collections and gastroscopy to exclude local ulceration or stenosis, based on the patient's clinical and laboratory status. We did not specifically attempt to measure or quantify bile in the reflux (e.g., bilirubin levels) in this study.

Patients were allowed to sip tea and water six hours postoperatively. Following NGT removal, and upon tolerance, their diet was gradually advanced from clear fluids to solid, easily chewable, low-fat, and low-fiber foods. Parenteral nutrition was discontinued once patients could tolerate adequate amounts of at least soft diet.

### Statistical analysis

Variables were tested for normality using histograms and Shapiro-Wilk tests. Normal distributions were expressed as means and standard deviations, and compared using *t*-tests or ANOVA. Non-normal distributions were expressed as median and inter-quartile ranges (IQR), and compared using Wilcoxon rank-sum tests or Kruskal-Wallis tests. Categorical variables were compared using Fisher's exact tests or Chi-squared tests. The association of potential risk factors with relevant postoperative DGE was examined using univariable logistic regression. Due to the limited number of events (DGE grade B or C), multivariable logistic regression was not conducted. The effect of a Braun enteroenterostomy following Billroth-II reconstruction was explored in a subgroup-analysis.

The statistical analysis was performed using R version 4.2.1 (2022-06-23) within RStudio version 2022.07.1 + 554. Used packages are acknowledged in Data Supplement 1. The significance-level was set at 0.05, and all tests were conducted two-sided.

## Results

### Patients and procedures

**Fig. 1:**
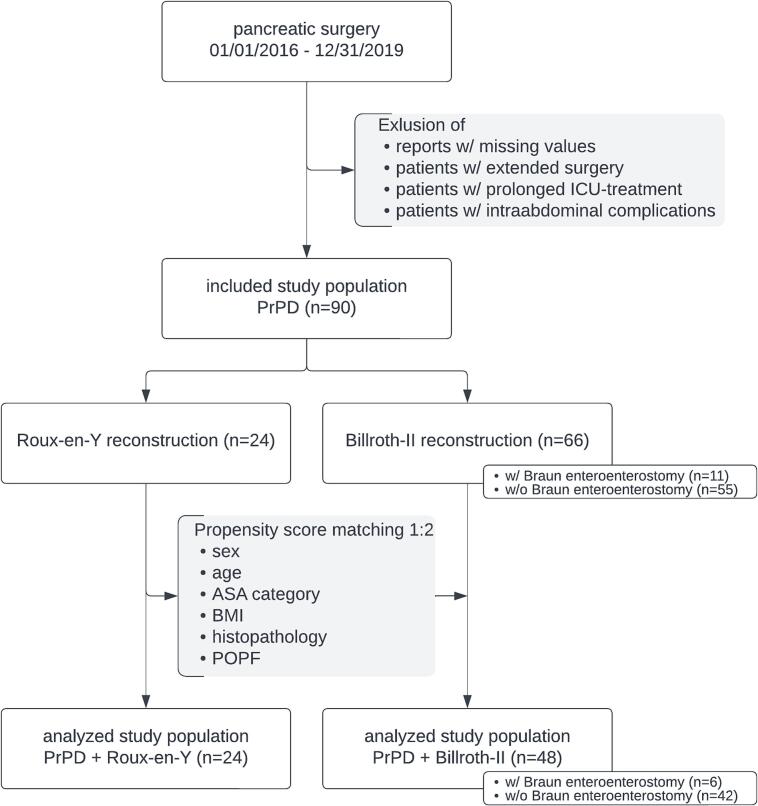
Study profile. Abbreviations: ASA, ASA physical status classification system; BMI, body mass index; ICU, intensive care unit; POPF, postoperative pancreatic fistula; PrPD, pylorus-resecting partial pancreatoduodenectomy; w/, with; w/o, without.

Among the included patients, 24 underwent PrPD followed by Roux-en-Y reconstruction. These patients were matched with 48 patients who underwent PrPD followed by single-looped, omega-shaped Billroth-II reconstruction (Fig. 1). Both groups were well-balanced regarding the matching criteria (Table 1). All procedures were performed by one of six surgeons, with each surgeon performing both types of reconstruction (cases per surgeon: Roux-en-Y 2 [8.3 %], 3 [12.5 %], 1 [4.2 %], 3 [12.5 %], 1 [4.2 %], 14 [58.3 %] vs. Billroth-II 4 [8.3 %], 8 [16.7 %], 1 [2.1 %], 13 [27.1 %], 2 [4.2 %], 20 [41.7 %]; *P* = 0.661).

**Table 1:** Patients' characteristics.

	PrPD with Roux-en-Y reconstruction (*n* = 24)	PrPD with Billroth-II reconstruction (*n* = 48)	*P*-Value
Age, years	72 [64 to 78]	72 [61 to 78]	0.886
Sex			
Female	15 (62.5 %)	30 (62.5 %)	1.000
Male	9 (37.5 %)	18 (37.5 %)	
ASA			
1/2	5 (20.8 %)	9 (18.8 %)	1.000
3/4	19 (79.2 %)	39 (81.3 %)	
BMI, kg/m^2^	24.6 [23.3 to 26.0]	24.1 [21.5 to 26.7]	0.272
Histopathology			
Benign	2 (8.3 %)	4 (8.3 %)	1.000
Malign	22 (91.7 %)	44 (91.7 %)	
Histopathology			
PDAC	13 (54.2 %)	34 (70.8 %)	0.159
pNET	–	3 (6.3 %)	
Distal CCA	1 (4.2 %)	3 (6.3 %)	
Papillary Ca	6 (25.0 %)	3 (6.3 %)	
Duodenal Ca	1 (4.2 %)	1 (2.1 %)	
Acinar cell Ca	1 (4.2 %)	–	
CP	2 (8.3 %)	2 (4.2 %)	
IPMN	–	1 (2.1 %)	
Papillary adenoma	–	1 (2.1 %)	
POPF			
None	15 (62.5 %)	36 (75.0 %)	0.286
BL	9 (37.5 %)	12 (25.0 %)	

Continuous data are presented as medians [interquartile range], and categorical data are shown as absolute values (percentages). *P*-values were derived from Fisher's exact test, Chi-squared test, or Kruskal-Wallis test.

Abbreviations: ASA, ASA physical status classification system; BL, biochemical leak; BMI, body mass index; Ca, carcinoma; distal CCA, distal cholangiocarcinoma; CP, chronic pancreatitis; IPMN, intraductal papillary mucinous neoplasm; IQR, interquartile range; PDAC, pancreatic ductal adenocarcinoma; pNET, pancreatic neuroendocrine tumor; POPF, postoperative pancreatic fistula; PrPD, pylorus-resecting partial pancreatoduodenectomy.

### Delayed gastric emptying

**Fig. 2:**
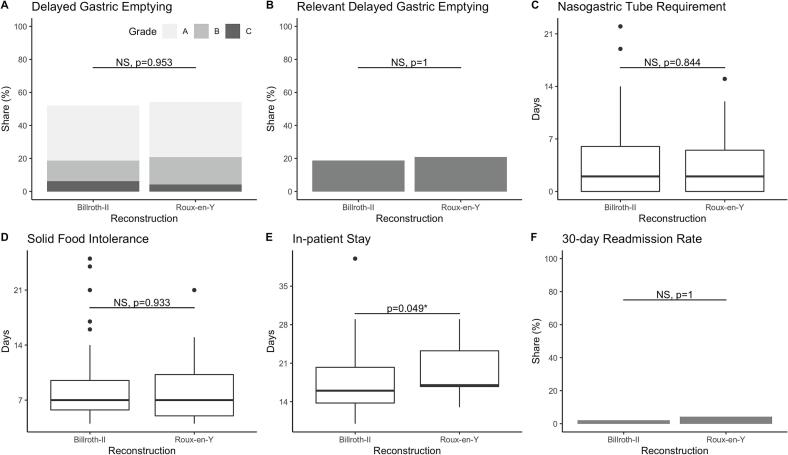
Outcomes. Outcomes of patients who underwent pylorus-resecting partial pancreatoduodenectomy (PrPD) with single-looped, omega-shaped Billroth-II reconstruction vs. Roux-en-Y reconstruction: A) delayed gastric emptying (DGE) by grade, B) DGE categorized as relevant (DGE grade B or C), C) duration of nasogastric tube requirement, D) time to tolerance of solid food, E) length of hospital stay, F) rate of 30-day readmissions. Significance of differences was tested using the Wilcoxon Rank Sum test or Fisher's exact test. Significant differences are indicated by an asterisk (*), while non-significant differences are denoted as NS (not significant). Abbreviations: DGE, delayed gastric emptying; PrPD, pylorus-resecting pancreatoduodenectomy; NS, not significant.

In this study population, 38 out of 72 patients (52.8 %) developed DGE. The rate of relevant DGE (grade B or C) did not differ significantly between patients who underwent PrPD followed by Roux-en-Y or Billroth-II reconstruction (5/24, 20.8 % vs. 9/48, 18.8 %; *P* = 1.000; Table 2, Fig. 2A). The severity of DGE was also similar between the two groups (no DGE, grade A, grade B and grade C: 11 [45.8 %], 8 [33.3 %], 4 [16.7 %] and 1 [4.2 %] vs. 23 [47.9 %], 16 [33.3 %], 6 [12.5 %] and 3 [6.3 %]; *P* = 0.953; Table 2, Fig. 2B). Both groups required NGT for similar durations (Table 2, Fig. 2C) and tolerated solid food at comparable times (Table 2, Fig. 2D). Patients who underwent Billroth-II reconstruction were discharged earlier, although there was high variability within the groups (median 16 days [IQR 13.7 to 20.3] vs. 17 [16.7 to 23.3]; *P* = 0.049; Table 2, Fig. 2E). The 30-day readmission rate was low and did not differ significantly between the groups (Table 2, Fig. 2F).

**Table 2:** Outcomes.

	PrPD with Roux-en-Y reconstruction (*n* = 24)	PrPD with Billroth-II reconstruction (*n* = 48)	*P*-value
DGE			
None	11 (45.8 %)	23 (47.9 %)	0.953
Grade A	8 (33.3 %)	16 (33.3 %)	
Grade B	4 (16.7 %)	6 (12.5 %)	
Grade C	1 (4.2 %)	3 (6.3 %)	
DGE			
Non-relevant	19 (79.2 %)	39 (81.3 %)	1.000
Relevant	5 (20.8 %)	9 (18.8 %)	
NGT requirement, days	2.0 [0.0 to 5.5]	2.0 [0.0 to 6.0]	0.844
Solid food intolerance, days	7.0 [5.0 to 10.3]	7.0 [5.7 to 9.5]	0.933
In-patient stay, days	17.0 [16.7 to 23.3]	16.0 [13.7 to 20.3]	0.049
30-day readmission	1 (4.2 %)	1 (2.1 %)	1.000

Continuous data are presented as medians [interquartile range], and categorical data are shown as absolute values (percentages). P-values were derived from Fisher's exact test, Chi-squared test, or Kruskal-Wallis test.

Abbreviations: DGE, delayed gastric emptying; IQR, interquartile range; NGT, nasogastric tube; PrPD, pylorus-resecting partial pancreatoduodenectomy.

In univariable logistic regression, the reconstruction method did not influence the rate of relevant DGE (OR 1.47 [95 % CI 0.42 to 4.72]; *P* = 0.524). However, older patients (OR 1.08 [1.02 to 1.17]; *P* = 0.030) and those with postoperative pancreatic biochemical leak (OR 4.98 [1.55 to 16.55]; *P* = 0.007) were more likely to develop relevant DGE (Table 3).

**Table 3:** Risk of relevant DGE.

Variable	Unit/category		Univariable logistic regression	
			OR [95 % CI]	*P*-value
Age	Years		1.08 [1.02 to 1.17]	0.030*
Sex	Female	vs. Male	0.46 [0.13 to 1.43]	0.193
ASA	1/2	vs. 3/4	0.69 [0.20 to 2.75]	0.565
BMI	kg/m^2^		1.13 [0.98 to 1.30]	0.087
Histopathology	Malign	vs. Benign	1.00 [0.23 to 7.00]	1.000
POPF	None	vs. BL	4.98 [1.55 to 16.55]	0.007*
Reconstruction	Billroth-II	vs. Roux-en-Y	1.47 [0.42 to 4.72]	0.524

Predictors of relevant delayed gastric emptying (DGE), defined as DGE grade B or C, derived from univariable logistic regression. Significance is indicated by an asterisk (*).

Abbreviations: ASA, ASA physical status classification system; BL, biochemical leak; BMI, body mass index; CI, confidence interval; OR, odds ratio; POPF, postoperative pancreatic fistula.

### Braun enteroenterostomy

In 6 out of 48 patients (12.5 %) who underwent Billroth-II reconstruction, an additional Braun enteroenterostomy was performed. In this subgroup, the frequency and severity of DGE were comparable to those in patients who did not receive a Braun enteroenterostomy (no DGE, grade A, grade B and grade C: 1 [16.7 %], 4 [66.7 %], 0 [0 %] and 1 [16.7 %] vs. 22 [52.4 %], 12 [28.6 %], 6 [14.3 %] and 2 [4.8 %]; *P* = 0.120). Similarly, the results were comparable to those in patients who underwent Roux-en-Y reconstruction (1 [16.7 %], 4 [66.7 %], 0 [0 %] and 1 [16.7 %] vs. 11 [45.8 %], 8 [33.3 %], 4 [16.7 %] and 1 [4.2 %]; *P* = 0.208).

## Discussion

This study investigated the rate and severity of primary delayed gastric emptying (DGE) in patients who underwent pylorus-resecting partial pancreatoduodenectomy (PrPD) followed by either Roux-en-Y or single looped, omega-shaped Billroth-II reconstruction. We hypothesized that resecting the pylorus might reduce DGE, but that potential advantages could be counterbalanced by an increased probability of biliary reflux. A Roux-en-Y reconstruction, instead of Billroth-II, might prevent this and thus reduce DGE.

In this study, 52.8 % of patients developed DGE. This rate is higher than that reported in a recent registry-based analysis in a comparable setting [6], but it is consistent with rates observed in other high-volume centers [5,25]. Also, most cases of DGE in the current study were classified as type A. The incidence and severity of DGE did not differ significantly between patients who underwent PrPD followed by Roux-en-Y reconstruction and those who underwent PrPD followed by Billroth-II reconstruction. Specifically, the duration of NGT use and the resumption of oral intake occurred within similar time frames for both groups. Although patients who underwent Billroth-II reconstruction were discharged earlier, the confidence intervals for these data were wide. It is important to note that hospital stay durations vary between countries due to differing patient expectations and healthcare structures.

In the literature, the optimal method of reconstruction following PrPD remains under debate: Shimoda et al. found a reduced rate of DGE after Billroth-II reconstruction (5.7 % vs. 20.4 %; *P* = 0.028) [26]. On the other hand, Barakat et al. and Ben-Ishay et al. reported an increased risk of DGE after Billroth-II compared to modified Roux-en-Y reconstruction (57.0 % vs. 10.2 %; *P* < 0.001 and 59.1 % vs. 15.4 %; *P* = 0.001) [27,28]. Additionally, studies by Busquets et al., Herrera et al. and Glowka et al. did not find significant differences in DGE rates between Billroth-II and Roux-en-Y reconstructions (45.0 % vs. 45.0 %; *P* = 1.000, and 25.0 % vs. 15.6 %; *P* = 0.350, and 62.8 % vs. 54.4 %; *P* = 0.272) [25,29,30]. However, in one study, Billroth-II reconstruction was performed without a Braun enteroenterostomy [29], and in two other studies, the pancreatoenteric anastomosis was constructed by pancreatogastrostomy [25,30], both potentially affecting the stomach's mucosa and causing DGE-like symptoms. A meta-analysis attempted to compare Billroth-II and Roux-en-Y reconstructions and reported a reduction of DGE after Billroth-II [31]. However, the definition of Roux-en-Y reconstruction varied among the included trials, with two of three trials isolating the pancreatojejunostomy in one limb from the hepaticojejunostomy and gastroenterostomy in the other.

Two network meta-analyses attempted to integrate results from various studies. Both Kamarajah et al. and Varghese et al. concluded that antecolic Billroth-II reconstruction is associated with the lowest rate of DGE [32,33]. According to Varghese et al., pylorus resection and Braun enteroenterostomy further reduce the risk of DGE [32]. However, both meta-analyses included patients who underwent pylorus-preserving partial pancreatoduodenectomy, and their recommendations are not specific for the optimal reconstruction after PrPD.

In summary, despite multiple trials and meta-analyses, the evidence regarding the optimal method of reconstruction after PrPD remains low, and findings are inconclusive. Technical variations in resection and reconstruction, as well as heterogeneous patient populations, compromise the comparability of studies. Furthermore, DGE is more likely to be caused secondarily by intraabdominal complications than primarily by a specific method of resection or reconstruction [10,16,17,19,22].

To address this, we excluded all patients with any intraabdominal complication (e.g., POPF grade B or C, postpancreatectomy hemorrhage) or prolonged ICU treatment due to non-surgical complications. We also considered known risk factors for DGE in the propensity score matching. Despite these measures, DGE rates associated with Roux-en-Y and Billroth-II reconstruction after PrPD were comparable in this study, and the reconstruction method did not significantly influence the rate of relevant DGE in logistic regression. Instead, consistent with previous studies, relevant DGE was more likely in older patients and in those with pancreatic biochemical leak [[16], [17], [18],^25^].

This study must be interpreted in the context of its design: The method of reconstruction was not randomized but determined at the surgeon's discretion. However, pancreatic surgery at our department is highly standardized, with both institutional and individual surgeon volumes being high. It should be noted that some patients who underwent Billroth-II reconstruction also had an Braun enteroenterostomy, which may have reduced biliary reflux and DGE in this group. Despite this, our subgroup analysis did not show a significant difference in the rate and severity of DGE between groups. While Billroth-II reconstruction without Braun enteroenterostomy is common practice following partial pancreatododenectomy [9,34], some meta-analyses suggest the contrary [[35], [36], [37]], especially after pylorus resection [25,38]. Based on these data, our current practice has evolved to favor Roux-en-Y reconstruction or Billroth-II reconstruction plus Braun enteroenterostomy following PrPD, and this approach has been implemented in the *PyloResPres* registry study [21].

The number of patients analyzed in this study is limited, which might result in the study being underpowered to detect a difference between the reconstruction methods. However, the confidence intervals do not suggest a relevant difference. Larger, prospective trials, such as the ongoing *PyloResPres* trial, are necessary to determine whether surgical alterations can influence DGE [21]. Until then, this study provides effect estimates useful for sample size calculations in future research.

## Conclusion

After PrPD, Roux-en-Y reconstruction was not associated with a reduced incidence or severity of DGE compared to Billroth-II reconstruction, despite its potential to prevent biliary reflux. Our findings indicate that even after excluding patients with significant intraabdominal complications, minor complications (such as pancreatic biochemical leak) and patient-specific factors (such as age) play a more substantial role in the development of DGE than the choice of surgical technique. Consequently, our findings align with previous research, suggesting that primary DGE is less relevant than DGE secondary to intraabdominal complications [17,19]. Therefore, focusing on minimizing these complications may be the most effective strategy for reducing DGE.

## Funding sources

This study was funded by the Department of General, Visceral and Transplantation Surgery, University Hospital, 10.13039/501100005722LMU Munich. None of the authors received a grant or specific financial or non-financial support related to this study.

## Ethical approval

This study was approved by the institutional review board of the medical faculty of LMU University (22-0951) and registered at the Clinical Study Center of the LMU University Hospital (100230).

## CRediT authorship contribution statement

**Felix O. Hofmann:** Writing – original draft, Visualization, Project administration, Methodology, Investigation, Formal analysis, Data curation, Conceptualization. **Victoria S. Engelstädter:** Writing – review & editing, Investigation, Data curation. **Ughur Aghamaliyev:** Writing – review & editing, Investigation, Data curation. **Mathilda M. Knoblauch:** Writing – review & editing, Investigation, Data curation. **Elise Pretzsch:** Writing – review & editing, Investigation, Data curation. **Maximilian Weniger:** Writing – original draft, Supervision, Methodology, Formal analysis, Conceptualization. **Jan G. D'Haese:** Writing – review & editing, Supervision, Conceptualization. **Bernhard W. Renz:** Writing – review & editing, Supervision, Conceptualization. **Jens Werner:** Writing – review & editing, Supervision, Conceptualization. **Matthias Ilmer:** Writing – review & editing, Supervision, Resources, Project administration, Conceptualization.

## Declaration of competing interest

None of the authors declared a conflict of interest.

## Appendix A Supplementary data

Supplementary material Image 1
