# Supplementary material for: Primary delayed gastric emptying after pylorus-resecting pancreatoduodenectomy: A matched-pair comparison of Roux-en-Y vs. Billroth-II reconstruction
Source: Surg Open Sci. 2024 Oct 31;22:46–52. doi: 10.1016/j.sopen.2024.10.005 (PMC11582468; doi:10.1016/j.sopen.2024.10.005)
Supplement: Supplementary file 1 — Supplementary material [file mmc1.pdf]

Primary Delayed Gastric Emptying after Pylorus-Resecting Pancreatoduodenectomy: A Matched-Pair Comparison of Roux-en-Y vs. Billroth-II Reconstruction

Data Supplement 1

Software

The statistical analysis was performed using R version 4.2.1 (2022-06-23) within RStudio version 2022.07.1+554. Used packages are listed below.

Package	Version	Purpose
data.table	1.14.5	Data management
MatchIt	4.4.0	Propensity Score Matching
ggplot2	3.3.6	Visualization
cowplot	1.1.1	Visualization
ggsignif	0.6.4	Visualization

Table 1: List of the used packages.
